# MRI-based atrophy subtypes in a young memory clinic cohort: associations with clinical and biomarker profiles

**DOI:** 10.1186/s13195-026-01972-2

**Published:** 2026-02-10

**Authors:** Alessandro Zilioli, Rosaleena Mohanty, Anna Rosenberg, Anna Matton, Tobias Granberg, Göran Hagman, Marco Spallazzi, Daniel Ferreira, Miia Kivipelto, Eric Westman

**Affiliations:** 1 Department of Neurology, University-Hospital of Parma, Parma, Italy; 2 Division of Clinical Geriatrics, Center for Alzheimer Research, Department of Neurobiology, Care Sciences and Society, Karolinska Institutet, Huddinge, Stockholm, Sweden; 3 Department of Public Health, Finnish Institute for Health and Welfare, Helsinki, Finland; 4 Ageing Epidemiology Research Unit, School of Public Health, Imperial College London, London, UK; 5 Department of Clinical Neuroscience, Karolinska Institutet, Stockholm, Sweden; 6 Department of Neuroradiology, Karolinska University Hospital, Stockholm, Sweden; 7 Theme Inflammation and Aging, Karolinska University Hospital, Stockholm, Sweden; 8 Facultad de Ciencias de la Salud, Universidad Fernando Pessoa Canarias, Las Palmas, España; 9 Institute of Public Health and Clinical Nutrition, University of Eastern Finland, Kuopio, Finland

**Keywords:** Alzheimer’s disease, Biological subtypes, Magnetic resonance imaging, Cerebrospinal fluid biomarkers

## Abstract

**Background:**

Brain atrophy subtypes are increasingly recognized in Alzheimer’s disease (AD) dementia. However, their relevance across the real-world memory clinic spectrum, from subjective cognitive impairment (SCI) and mild cognitive impairment (MCI) to AD and non-AD dementias, remains unclear. This cross-sectional study aimed to identify MRI-based atrophy subtypes in a relatively young memory clinic and examine associations with demographic, cerebrospinal fluid (CSF) biomarkers, and cerebrovascular burden to inform precision medicine approaches.

**Methods:**

We included all consecutive patients (SCI to dementia), evaluated at the Karolinska University-Hospital Memory Clinic (Stockholm, Sweden) between 2018 and 2023 with available clinical and 3T MRI data. Subtypes were defined using FreeSurfer-derived volumetric measures and a validated algorithm combining categorical classification (typical, limbic predominant, cortical predominant, minimal atrophy) with continuous indices of typicality (cortical predominant–limbic predominant) and severity (minimal atrophy–typical). Demographics, cognitive profiles, *APOE* ε4 status, CSF biomarkers (Aβ42, Aβ42/40, phosphorylated [p]-tau181, total tau, neurofilament light chain [NFL]), and cerebrovascular burden were compared across subtypes. Analyses were replicated in Aβ-positive individuals and those eligible for anti-Aβ therapy.

**Results:**

Among 809 patients (median age 60.0 years [interquartile-range 56.0–63.0], 56.1% female), 38.2% had SCI, 44.4% MCI, and 17.4% dementia. CSF biomarkers were available in 596 (73.7%).

Limbic predominant and typical subtypes had more males (59.3% and 50.0%, respectively; group-wise p < 0.001), higher APOE ε4 frequency (47.7% and 41.0%, p = 0.02), greater cerebrovascular burden, and poorer memory. These subtypes were more often Aβ positive (46.1% and 46.5%, p = 0.01). A cortical predominant pattern was frequent in females (66.0%, p < 0.001), while minimal atrophy was associated with milder cognitive impairment (49.0% SCI, 45.5% MCI) and higher depressive symptoms. In Aβ-positive patients (n = 231), typical and limbic subtypes had higher p-tau181 (median: 83.0 and 84.5 pg/mL, respectively; p < 0.001), NFL (1120.0 and 1125.0 pg/mL, p < 0.001), and lower Aβ42/40 ratios (0.051 and 0.049, p = 0.02). Findings remained consistent across continuous atrophy measures and in the 14.9% (n = 89) eligible for anti-Aβ therapy.

**Discussion:**

MRI-based atrophy subtypes exhibit distinct clinical and biomarker profiles, consistently observed in Aβ-positive and anti-Aβ-therapy-eligible patients. These findings support their diagnostic utility in memory clinics and relevance for biologically targeted AD trials.

**Supplementary Information:**

The online version contains supplementary material available at 10.1186/s13195-026-01972-2.

## Introduction

Alzheimer’s disease (AD), defined by amyloid β (Aβ) and hyperphosphorylated tau (p-tau) neurofibrillary tangles (NFTs), is the leading cause of dementia [1]. Traditionally considered a single clinicopathological entity, AD is now recognized as a heterogeneous disorder [2]. In addition to the typical amnestic presentation, atypical variants such as frontal (behavioral), posterior cortical atrophy (PCA; visuospatial), and primary progressive aphasia (PPA; language) have been extensively described [3], particularly in early-onset AD (EOAD; onset traditionally defined < 65 years) [4].

Beyond clinical phenotypes, AD also shows substantial neuropathological heterogeneity. While Aβ deposition follows a stereotypical cortico-caudal spread (as described by Thal staging) [5], NFT distribution is more variable. Postmortem studies have identified three main subtypes based on regional NFT burden: limbic predominant (with predominant involvement of medial temporal areas), cortical predominant (primarily affecting cortical regions), and typical (involving both medial temporal and cortical areas) [6]. These patterns have been translated into in vivo MRI-based classifications reflecting regional atrophy distributions [7]. Converging postmortem and in vivo imaging evidence indicates that regional tau burden is more closely linked to patterns of cortical atrophy than amyloid deposition, with tau topography spatially and temporally colocalizing with neurodegeneration, thereby providing a biological substrate for MRI-based atrophy subtypes [8]. In addition to these patterns, a fourth, the minimal atrophy subtype, characterized by limited or absent gray matter loss despite biomarker evidence of AD pathology, has been consistently described across independent cohorts [9, 10].

A recent meta-analysis confirmed subtype-related differences in demographics, APOE ε4 genotype distribution, and biomarker profiles [11]. Typical AD was most frequent and associated with balanced medial temporal and temporoparietal atrophy and amnestic presentations in older APOE ε4 carriers [6, 7, 12]. The limbic-predominant subtype showed pronounced hippocampal and entorhinal atrophy, slower progression, and frequent comorbidities such as TDP-43 proteinopathy [6, 7, 13]. Cortical predominant AD was characterized by parietal, occipital, and dorsolateral frontal thinning, relative hippocampal preservation, non-amnestic presentations, and an aggressive course with greater tau burden [6, 14, 15]. The minimal atrophy subtype comprised patients fulfilling AD biomarker criteria but showing only subtle atrophy changes, often with lower educational attainment and potentially reflecting early disease or mixed pathologies [9, 16, 17].

More recently, these subtypes have also consistently been observed using Tau-and FDG-PET [18–20]. Although subtypes from different neuroimaging modalities are very similar at the group level, differences may occur at the individual level, potentially reflecting the temporal ordering of biomarkers or the potential role of co-pathologies [20, 21].

Most MRI studies focused on late-onset AD at the dementia stage, while data on younger individuals and earlier phases such as mild cognitive impairment (MCI) or subjective cognitive impairment (SCI) remain limited [6–8, 10, 18]. This gap is clinically relevant, as the therapeutic window for anti-Aβ interventions may lie in these stages [22].

Moreover, available evidence mostly derives from highly selected cohorts such as the Alzheimer’s Disease Neuroimaging Initiative (ADNI) [23, 24], which apply stringent inclusion criteria (e.g., excluding cerebrovascular comorbidities), limiting generalizability to clinical practice. Different MRI subtyping methods have also been proposed, underscoring the need for harmonization [25]. Further studies are also needed to assess whether MRI-based subtypes can capture biological and clinical heterogeneity in unselected memory clinic populations, where non-amnestic symptoms, atypical phenotypes, and non-AD etiologies are common [26].

We therefore aimed to characterize MRI-based atrophy subtypes in a naturalistic, relatively young memory clinic cohort using a neuropathologically validated MRI-subtyping approach. We examined subtype-related differences in demographic and clinical features, APOE ε4 status, cerebrospinal fluid (CSF) biomarkers, and cerebrovascular burden. Among patients with available CSF data, we further stratified by Aβ status to determine whether observed subtype patterns were primarily driven by AD pathology. Lastly, we assessed whether subtype-specific differences remained detectable in individuals meeting eligibility criteria for anti-Aβ therapy, as defined by recently proposed Lecanemab guidelines [27], with the aim of advancing biologically informed precision medicine strategies for AD. We hypothesized that MRI-based atrophy subtypes capture biologically meaningful heterogeneity across the memory clinic spectrum, even in a relatively young and clinically unselected population. We expected that the limbic-predominant and typical subtypes, representing the most common atrophy patterns in AD, would be associated with a greater burden of AD-related pathology, reflected by higher amyloid and tau positivity, compared with the cortical predominant and minimal atrophy subtypes. Further, we hypothesized that these subtype-related differences would be evident in Aβ-positive individuals and in patients eligible for anti-Aβ therapy, supporting the presence of distinct biological underpinnings within the AD spectrum, while being attenuated or absent in Aβ-negative patients. Finally, we expected that continuous measures of atrophy typicality and severity would show graded associations with CSF biomarkers of amyloid, tau, and neurodegeneration.

## Methods 

### Standard protocol approvals, registrations, and patient consents

The Karolinska University Hospital electronic database and biobank for clinical research (GEDOC) and this study have received ethical approval (Regional Ethical Review Board in Sweden; Dnr 2011/1987-31/4 and 2020–06484).

All procedures were conducted in accordance with ethical standards and with written informed consent from all participants.

### Participants and study design

This study was performed at the Karolinska University Hospital in collaboration between the Medical Unit Aging Memory Clinic, Karolinska University Hospital and the Division of Clinical Geriatrics, Center for Alzheimer Research, Department of Neurobiology, Care Sciences, and Society, Karolinska Institutet.

This specialized outpatient clinic evaluates individuals with cognitive complaints referred by general practitioners in primary and occupational health services in northern Stockholm and individuals under 70 years of age referred from the broader Stockholm region.

All diagnostic procedures were completed within one week, following a “fast track model” described previously [28].

The standardized diagnostic protocol included neurological and medical examinations, detailed history-taking from patients and informants, comprehensive neuropsychological testing, blood chemistry, 3 T MRI, APOE genotyping, and CSF biomarker analysis (Aβ42, Aβ42/40 ratio, phosphorylated tau [p-tau181], total tau [t-tau], and neurofilament light chain [NFL]). Additional assessments were conducted when clinically indicated. Diagnoses were established according to DSM-5 criteria and ICD-10 coding.

Consecutive patients with a first clinical visit between April 2018 and November 2023 were included. To reflect routine clinical practice, inclusion criteria were intentionally non-restrictive, requiring only cognitive complaints and availability of a 3 T MRI. Exclusion criteria included unstable medical or psychiatric conditions (e.g., recent cancer, major depression, or acute cardiovascular/cerebrovascular events) or recent initiation of related treatments. A total of 809 patients completed MRI and were included for subtype classification and descriptive analyses; 596 (73.6%) also had CSF data and were stratified into Aβ-positive and Aβ-negative groups.

MRI-based subtyping was applied to the entire cohort, regardless of Aβ status, to investigate subtype-related differences in demographic, clinical, APOE ε4, cerebrovascular, and biomarker profiles. Subgroup analyses were subsequently conducted in Aβ-positive and Aβ-negative patients to assess whether atrophy patterns were biologically meaningful in the presence of AD pathology and whether analogous patterns could be identified in Aβ-negative individuals. Lastly, we assessed whether subtype-specific differences remained detectable in individuals meeting eligibility criteria for anti-Aβ therapy, as defined by recently proposed Lecanemab guidelines [27].

Although the Memory Clinic primarily serves individuals younger than 65 years, representing a relatively young cohort, no age-based cut-off was applied in the main analyses to maintain a naturalistic, real-world design. All primary analyses were replicated in the subgroup aged < 65 years and are presented in the Supplementary materials.

### Neuroimaging assessment

MRI scans were acquired on a 3 T Discovery MR750 system (GE Healthcare, Milwaukee, WI) using a standard clinical head coil. The protocol included multiple high-resolution sequences: a sagittal 3D T1-weighted BRAVO sequence (1 mm isotropic; TR = 8.16 ms; TE = 3.18 ms), 3D T2-weighted FLAIR CUBE (1.2 mm; TR = 8000 ms; TE = 116 ms), axial T2-weighted PROPELLER (4 mm; TR = 4000 ms; TE = 86 ms), axial diffusion-weighted imaging (DWI; 4 mm; TR = 3501 ms; TE = 62 ms), and axial susceptibility-weighted imaging (SWI 3D; 2 mm; TR = 30 ms; TE = 20 ms).

The T1-weighted 3D BRAVO images were processed through the HiveDB system [29] using FreeSurfer 7.3.2 (http://surfer.nmr.mgh.harvard.edu/). Detailed quality control (QC), i.e. visual inspection was performed on the native images to exclude artifacts (incomplete coverage and motion artifacts etc.) according to previously published criteria. Further, QC was also performed on the FreeSurfer processing output to check for segmentation errors [30]. Automatic region of interest segmentation produced volumes in cortical and subcortical structures [31]. White matter (WM) hypointensities, normalized to intracranial volume (ICV), were used as a proxy for cerebrovascular burden within the FreeSurfer pipeline. WM hypointensities have been shown to exhibit high concordance with conventional WM hyperintensity volume measurements, while appearing to be more specific markers of WM structural integrity and less strongly associated with peri-inflammatory processes and other phenomena related to blood–brain barrier permeability [32].

In the clinical routine all images are assessed by experienced neuroradiologist using standardized visual ratings scales for medial temporal lobe atrophy (MTA), global cortical atrophy (GCA), and WM hypointensities [33]. Cerebral microbleeds were defined on SWI images according to the Standards for Reporting Vascular Changes (STRIVE) criteria (present or not present) [34].

### CSF analysis

CSF samples were collected under sterile conditions using polypropylene tubes (Medicarrier, art. no. 67741) and analyzed at the Karolinska University Hospital Laboratory. Prior to August 21, 2019, the concentrations of Aβ42, Aβ40, phosphorylated tau 181 (p-tau181), total tau (t-tau), and neurofilament light chain (NFL) were measured using commercially available Innotest sandwich enzyme-linked immunosorbent assays (Fujirebio Europe, Ghent, Belgium). From August 22, 2019, CSF samples were analyzed using the Lumipulse G-series (Fujirebio Europe, Ghent, Belgium), a fully automated chemiluminescent enzyme immunoassay platform. High concordance between the two methods has been reported [35]. Amyloid positivity (A + status) was defined using data-driven cut-offs: <0.60 (Innotest) and < 0.86 (Lumipulse) for the Aβ42/40 × 10 ratio [28]. As an additional and robust indicator of AD-related pathology, the t-tau/Aβ42 ratio was also calculated [36].

### Cognitive data

Cognitive assessment included the Montreal Cognitive Assessment (MoCA) [37] and/or Mini-Mental State Examination (MMSE) [38] to evaluate global cognitive function, the Rey Auditory Verbal Learning Test (RAVLT) for assessing memory through immediate and delayed recall [39], the Rey Complex Figure Test (copy and delayed recall) [40] to assess visuospatial abilities and short-term visual memory, and the coding subtest from the Wechsler Adult Intelligence Scale (WAIS), 4th edition, to evaluate attention and processing speed functions [41]. Depressive symptoms were screened using the Patient Health Questionnaire (PHQ-9) [42].

### Serum and genetic data

The following blood-based analytes were assessed using plasma or serum samples: haemoglobin, glycated haemoglobin (HbA1c), high-density lipoprotein cholesterol (HDL-C), total cholesterol, homocysteine, and thyroid-stimulating hormone. In addition, APOE ε4 genotype was determined, and individuals were classified as ε4 carriers if heterozygous or homozygous (+/− or +/+).

### Biological subtypes classification

Several methods have been applied to define biological subtypes of AD, highlighting the need for harmonization [25]. A new conceptual framework has been proposed to study disease heterogeneity along two continuous dimensions: typicality, ranging from limbic-predominant to cortical predominant, and severity, ranging from minimal atrophy to the typical subtype [11]. In our study, we evaluated the concepts of typicality and severity using both categorical and continuous approaches, as demonstrated in prior studies [26, 43]. We applied these subtyping methods to the entire cohort, irrespective of CSF biomarker status, to determine which MRI-based atrophy subtype was most strongly associated with Aβ positivity and whether subtype-related differences were also present among individuals without an AD biomarker profile.

We selected the hippocampal-to-cortical volume (HV/CTX) ratio as a proxy for typicality, as it is the only metric with histopathological validation. The HV/CTX ratio was calculated by dividing the average volume of the left and right hippocampi by the average volume of three cortical regions: (I) the middle frontal gyri, (II) the inferior parietal gyri, and (III) the superior temporal gyri [6].

In the categorical approach, patients below the 25th percentile (HV/CTX < 0.246) were classified as belonging to the limbic-predominant subtype, while those above the 75th percentile (HV/CTX > 0.286) were categorized as cortical predominant. We used the brain volume (BV)-to CSF index to define the severity for the remaining patients [44]. In the categorical approach, patients below the 50th percentile were characterized as typical (BV/CSF < 883.5), while those above the 50th percentile (BV/CSF > 883.5) were classified as having minimal atrophy.

### Eligibility for anti-Aβ treatment

Eligibility for anti-Aβ treatment was assessed using recently proposed appropriate use criteria for Lecanemab [27].

Specifically, inclusion criteria were as follows: (I) a clinical diagnosis of MCI due to AD or mild AD dementia, (II) MMSE scores between 22 and 30 or MoCA scores ranging from 17 to 30, (III) stable systemic illnesses and psychiatric conditions, (IV) a CSF profile consistent with AD, defined as A + status, (V) no anticoagulant treatment, and (VI) no MRI evidence of cerebral microbleeds.

### Statistical analysis

Data are presented as percentages for categorical variables and as medians with first and third quartiles for continuous variables. Group comparisons were performed using one-way ANOVA or ANCOVA for normally distributed data, the Kruskal–Wallis test for non-normally distributed data, and Fisher’s exact test for binary variables. Post hoc pairwise comparisons were conducted using Bonferroni correction for ANOVA/ANCOVA and Fisher’s exact test, and the Dwass–Steel–Critchlow–Fligner (DSCF) procedure for Kruskal–Wallis tests.

Cognitive differences across categorical subtypes were evaluated with adjustment for age, sex, and education. White matter hypointensity volumes were corrected for brain volume (BV). Cognitive and cerebrovascular outcomes were analysed as individual measures rather than composite scores to preserve domain-specific and pathophysiological interpretability. This approach allows the identification of differential cognitive profiles and distinct cerebrovascular mechanisms potentially associated with specific MRI-based atrophy subtypes.

For CSF biomarkers, adjustments for age, sex, or APOE ε4 carrier status were not applied, as the influence of these factors is not fully established [45], and will be evaluated in subsequent analyses. To account for potential methodological variability, CSF biomarker analyses were additionally repeated using a general linear model (GLM) including assay platform (e.g., Innotest, Lumipulse) as a covariate, which was shown not to alter the results of the analyses. The proportion of missing CSF data was comparable across MRI-based atrophy subtypes, with no evidence of subtype-specific pattern.

In addition, serum and CSF biomarkers were analyzed in relation to MRI-derived indices of typicality (HV/CTX) and severity (BV/CSF) treated as continuous variables. Given the non-normal distribution of biomarker measures, Spearman correlation matrices were used.

Statistical significance was set at *p* < 0.05, corrected for false discovery rate (FDR). Effect sizes were systematically reported to quantify the magnitude of group differences and associations, including eta squared (η²) for ANOVA/ANCOVA, epsilon squared (ε²) for Kruskal–Wallis tests, and Cramér’s V for categorical variables. For all effect size estimates, 95% confidence intervals were obtained using non-parametric bootstrap resampling. All analyses were performed using RStudio version 4.4.2.

## Results

### Study population characteristics

The cohort comprised 809 consecutive patients who underwent 3 T MRI with volumetric post-processing. Median age was 60.0 years (interquartile range [IQR]: 56.0–63.0), with 85.4% aged < 65 years. Females represented 56.1% of the sample. Median education was 13.0 years (IQR: 11.0–16.0), and median MoCA score was 24.0 (IQR: 20.0–26.0). Clinical diagnoses were mainly subjective cognitive impairment (SCI, 38.2%) or mild cognitive impairment (MCI, 44.4%). Most dementia cases with biological confirmation were of the Alzheimer’s disease type (64.9%). Non–AD diagnoses included non-AD neurodegenerative dementias (frontotemporal dementia, dementia with Lewy bodies, Parkinson’s disease dementia, progressive supranuclear palsy, and Creutzfeldt–Jakob disease; accounting for 15.2% of cases); vascular dementia (8.0%); other dementia-related conditions (e.g., alcohol-related cognitive disorder or primary psychiatric conditions, 2.9%); and dementia of unspecified etiology (9%).

### MRI-based atrophy subtype differences in the global cohort

**Fig. 1:**
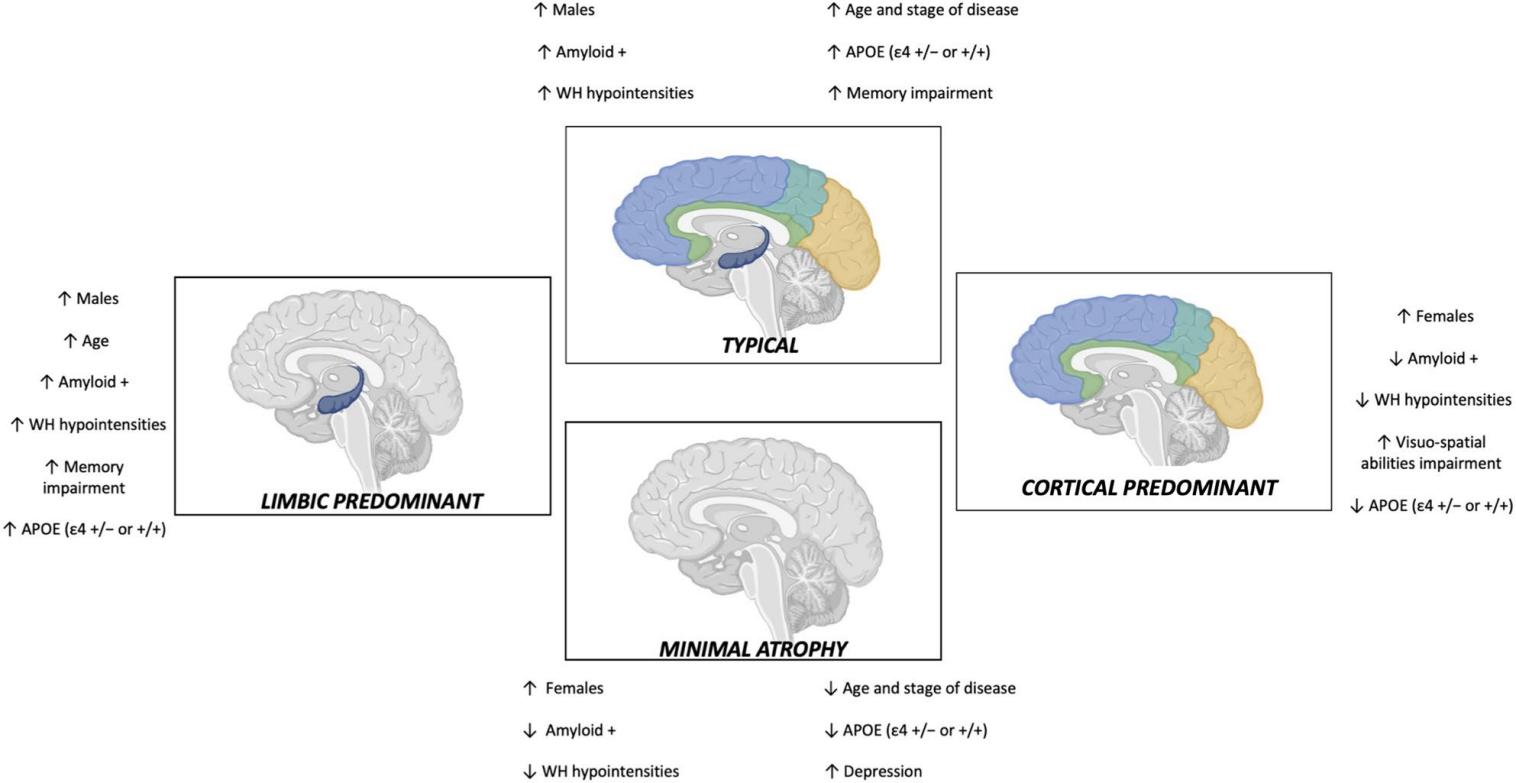
Summary of demographic, cognitive assessment, CSF, and vascular profiles among the MRI-based atrophy subtypes. On the horizontal axis, typicality (from limbic predominant to cortical predominant); on the vertical axis, severity (from minimal atrophy to typical)

Subtype characteristics are presented in Table 1; Fig. 1. Age differed significantly across subtypes (group-wise *p* < 0.001, ε² = 0.03; 95% CI, 0.01–0.06), being lowest in the minimal atrophy group (median: 58.0 years) and highest in the typical and limbic predominant subtypes (median: 61.0 years).

**Table 1:** Summary of the demographic, cognitive assessment, neuroimaging, and CSF biomarkers of the global cohort

	n	All	Cortical predominant	Limbic predominant	Typical	Minimal atrophy	Global P-value FDR-corrected and pairwise comparisons
Sample size	809	809	206	199	202	202	-
Demographic variables							
Age (years)	809	60.0 [56.0-63.0]	60.0 [55.0-63.0]	61.0 [57.0-64.0]	61.0 [57.0-64.0]	58.0 [55.0-62.0]	< 0.001^e, f^
Gender (female, %)	809	56.1%	66.0%	40.7%	50.0%	66.8%	< 0.001^a, b, e, f^
Education (years)	641	13.0 [11.0-16.0]	13.0 [12.0-15.0]	13.2 [11.6-16.0]	13.0 [11.0-16.0]	12.5 [11.0-16.0]	0.64
APOE (ε4 +/− or +/+, %)	706	45.8%	32%	47.7%	41.0%	39.1%	0.02^a, b, e^
Cognitive assessment							
MoCA	645	24.0 [20.0-26.0]	23.0 [19.5-26.0]	24.0 [21.0-26.0]	24.0 [17.5-26.0]	25.0 [22.0-27.0]	<0.001 ^c, f^
MMSE	480	27.0 [24.0-29.0]	26.0 [23.0-28.0]	27.6 [24.0-29.0]	27.0 [23.0-29.0]	27.0 [25.0-29.0]	0.04^f^
RAVLT immediate recall	488	42.5 [32.0-51.0]	44.0 [32.0-51.0]	39.0 [30.0-49.0]	38.0 [28.0-49.0]	47.0 [37.0-53.75]	< 0.001^e, f^
RAVLT delayed recall	488	9.0 [5.0-12.0]	10.0 [6.0-12.0]	7.0 [3.0-11.0]	8.0 [3.0-12.0]	11.0 [8.0-13.0]	< 0.001^a, e, f^
RCF copy	469	33.0 [30.0-34.0]	33.0 [30.0-34.0]	32.0 [29.5-34.0]	32.0 [31.0-34.0]	33.0 [31.0-34.0]	0.38
RCF delayed recall	469	15.0 [8.5-21.0]	15.0 [9.0-19.5]	14.0 [7.0-19.0]	14.0 [6.0-20.0]	18.0 [12.5-23.0]	< 0.001^e, f^
WAIS-IV coding	468	49.0 [38.0-61.0]	47.0 [34.2-58.7]	50.0 [41.0-61.0]	45.0 [34.0-59.0]	52.0 [4.0-64.5]	0.04 ^c^
PHQ-9	597	6.5 [3.0-11.0]	7.0 [2.3-12.0]	6.0 [2.0-11.0]	6.0 [2.0-9.7]	8.0 [4.0-13.0]	0.01^e, f^
Blood biomarkers Homocysteine	744	12.0 [10.0-15.0]	12.0 [9.9-14.0]	13.0 [11.0-15.5]	13.0 [11.0-16.0]	12.0 [9.9-14.0]	<0.001^a, b, e, f^
(µmol/L)							
LDL-cholesterol (mmol/L)	692	3.0 [2.4-3.7]	2.9 [2.3-3.6]	3.0 [2.4-3.6]	3.0 [2.4-3.7]	3.2 [2.6-3.8]	0.19
HbA1c (mmol/mol)	728	36.5 [34.0-39.0]	37.0 [34.5-39.0]	36.0 [34.0-39.0]	37.0 [34.0-39.0]	36.0 [34.0-39.0]	0.38
Neuroimaging biomarkers							
HV/CTX	809	0.26 [0.24-0.28]	0.30 [0.29-0.31]	0.22 [0.21-0.23]	0.26 [0.25-0.27]	0.26 [0.25-0.27]	< 0.001^a, b, c, d, e^
BV/CSF	809	883.6 [735.5-1026.8]	919.6 [795.5-1092.8]	831.5 [696.3-974.4]	736.8 [670.1-799.5]	1025.6 [960.9-1148.8]	< 0.001^a, b, c, d, e, f^
WM hypointensities (mm^3^)	809	1275.8 [761.8-2580.3]	967.2 [607.3-2053.7]	1785.6 [1012.6-3524.1]	1798.2 [1094.0-3235.6]	929.4 [636.2-1783.5]	< 0.001^a, b, e, f^
CSF biomarkers							
Aß42, pg/mL	596	906.5 [645.7-1192.5]	961.0 [724.0-1260.0]	827.0 [585.0-1010.0]	753.0 [569.5-1062.5]	1050.0[761.7-1277.5]	< 0.001^a, b, e, f^
Aß42/Aß40	596	0.09 [0.05-0.10]	0.09 [0.06-0.10]	0.08 [0.05-0.10]	0.07 [0.05-0.10]	0.09 [0.07-0.10]	< 0.001^a, b, e, f^
p-tau, pg/ml	596	42.0 [30.0-63.0]	38.0 [28.5-55.5]	46.0 [33.0-82.0]	44.0 [31.0-79.5]	40.5 [30.0-52.0]	< 0.001^a, b, e^
t-tau, pg/ml	596	293.5 [208.0-442.0]	266.0 [187.0-363.0]	319.0 [230.5-523.5]	320.5 [219.7-530.0]	289.0 [199.0-366.0]	< 0.001^a, b, e, f^
NFL, pg/ml	594	800.0 [610.0-1157.5]	740.0 [555.0-970.0]	960.0 [650.0-1360.0]	975.0 [710.0-1312.5]	700.0 [570.0-910.0]	< 0.001^a, b, e, f^
t-tau/Aß42	596	0.52 [0.40-0.73]	0.49 [0.37-0.65]	0.57 [0.47-0.81]	0.63 [0.44-0.83]	0.45 [0.37-0.62]	< 0.001^a, b, e, f^
Amyloid status (A+, count and %)	596	231 (38.8%)	51 (32.9%)	66 (46.1%)	67 (46.5%)	47 (30.5%)	0.01^e, f^
Disease stage							
SCI (%)	809	38.2%	41,2%	29.1%	33.6%	49%	< 0.001^c, e, f^
MCI (%)	809	44.4%	42.2%	50.7%	39.1%	45.5%	
Dementia (%)	809	17.4%	16.6%	20.2%	27.3%	5.5%	

All quantitative data are represented with median and 1st interquartile and 3rd interquartile [Q1-Q3]. Letters indicate significant pairwise comparisons, after Dwass-Steel-Critchlow-Fligner (DSCF) or Bonferroni correction

WM hypointensities are presented as “raw” data and normalized for the ICV to assess differences across subtypes

^a^Cortical predominant versus limbic predominant

^b^Cortical predominant versus typical

^c^Cortical predominant versus minimal atrophy

^d^Limbic predominant versus typical

^e^Limbic predominant versus minimal atrophy

^f^Typical versus minimal atrophy

Sex distribution also varied, with a higher proportion of females in the cortical predominant and minimal atrophy groups (66.0% and 66.8%, respectively; *p* < 0.001, Cramér’s V = 0.22; 95% CI 0.16–0.3*)*. APOE ε4 genotype (+/− or +/+) was more frequent in the limbic predominant and typical subtypes (47.7% and 41.0%, *p* = 0.02, Cramér’s V = 0.12; 95% CI, 0.04–0.19).

Cognitive profiles differed significantly, with MoCA scores being highest in the minimal atrophy group (median: 25.0) and lower in the typical and cortical predominant subtypes (median: 24.0 and 23.0, respectively).

The minimal atrophy subtype showed more pronounced depressive symptoms (median PHQ-9 score: 8.0; *p* = 0.01, η² = 0.02; 95% CI, 0.006–0.05). Typical and limbic predominant patients showed poorer long-term and visual memory performance, as assessed by RAVLT and RCF delayed recall (both *p* < 0.001; η² = 0.05, 95% CI 0.03–0.1, and η² = 0.05, 95% CI 0.02–0.1, respectively).

CSF profiles varied significantly: limbic predominant and typical subtypes showed lower Aβ42 (median: 827.0 and 753.0 pg/mL, respectively) and Aβ42/40 ratio (0.08 and 0.07), and higher p-tau (46.0 and 44.0 pg/mL), t-tau (319.0 and 320.5 pg/mL), NFL (960.0 and 975.0 pg/mL), and t-tau/Aβ42 ratio (0.57 and 0.63) compared to hippocampal-sparing and minimal atrophy (*p* < 0.001 and ε² > 0.03 for all). Aβ-positivity was most frequent in the typical (46.5%) and limbic predominant (46.1%) groups. WM hypointensity volumes and homocysteine levels were significantly higher in both the typical and limbic-predominant subtypes (both *p* < 0.001), with moderate effect sizes for WM hypointensities (ε² = 0.10, 95% CI 0.06–0.14) and small effect sizes for homocysteine (ε² = 0.02, 95% CI 0.004–0.05). Findings in patients < 65 years (*n* = 691) were consistent (Supplementary eTable 1).

### MRI-based atrophy subtype differences in the Aβ-positive cohort

Subtype characteristics for the Aβ-positive group (A + status, *n* = 231; 38.7% of patients with available CSF data) are summarized in Table 2. Most patients were diagnosed with MCI (46.8%) or AD-dementia (32.0%), while 21.2% had subjective cognitive impairment (SCI).

**Table 2:** Summary of the demographic, cognitive assessment, and neuroimaging and CSF biomarkers of the Aβ-positive cohort

	n	All	Cortical predominant	Limbic predominant	Typical	Minimal atrophy	Global P-value FDR-corrected and pairwise comparisons
Sample size	231	231	51	66	67	47	-
Demographic variables							
Age (years)	231	61.0 [58.0-64.0]	61.0 [58.0-64.0]	62.0 [58.3-64.0]	61.0 [57.0-64.0]	60.0 [57.0-63.0]	0.42
Gender (female, %)	231	57.6%	66.6%	45.4%	55.2%	68.0%	0.12
Education (years)	185	13.5 [12.0-16.0]	13.0 [12.0-15.7]	13.5 [12.0-16.0]	13.0 [11.5-16.0]	13.7 [11.2-16.3]	0.99
APOE (ε4 +/− or +/+, %)	225	68.4%	66.6%	69.6%	62.6%	68.0%	0.99
Cognitive assessment							
MoCA	195	22.0 [17.0-25.0]	21.0 [17.0-25.0]	23.0 [19.0-25.0]	18.0 [14.0-24.0]	24.0 [22.0-25.0]	< 0.001^d, f^
MMSE	147	26.0 [22.0-28.5]	24.0 [22.2-27.2]	28.0 [24.0-29.0]	24.0 [20.0-28.0]	27.0 [25.5-29.0]	0.04^f^
RAVLT immediate recall	142	35.0 [27.2-47.0]	33.5 [25.7-48.7]	33.5 [27.7-47.0]	31.0 [20.5-39.5]	44.0[33.0-51.0]	0.06
RAVLT delayed recall	142	7.0 [3.0-11.0]	7.0 [4.0-11.0]	4.5 [2.7-10.0]	4.0 [2.0-8.5]	9.0 [4.0-12.0]	0.07
RCF copy	133	33.0 [30.0-35.0]	31.0 [25.0-34.0]	33.7 [31.0-34.0]	32.5 [29.0-34.2]	33.5 [31.0-35.0]	0.38
RCF delayed recall	133	12.0 [6.0-18.0]	12.0 [3.0-18.5]	11.0 [7.8-16.7]	7.0 [3.2-15.6]	14.2 [10.3-18.0]	0.07
WAIS-IV coding	135	47.0 [34.5-58.0]	39.0 [26.0-49.0]	51.0 [39.0-60.5]	43.5 [31.5-52.0]	49.0 [41.7-63.2]	0.12
PHQ-9	166	5.0 [2.0-10.0]	6.0 [2.0-11.2]	4.0 [2.0-9.0]	4.0 [1.8-7.5]	8.0 [4.0-13.0]	0.02^e, f^
Blood biomarkers							
Homocysteine (µmol/L)	223	13.0 [10.0-16.0]	13.0 [10.0-15.0]	13.0 [12.0-16.2]	13.0 [11.0-16.0]	12.0 [9.9-15.0]	0.38
LDL-cholesterol (mmol/L)	208	3.2 [2.5-3.8]	2.8 [2.4-3.5]	3.2 [2.6-3.8]	3.1 [2.6-3.7]	3.3 [2.7-4.0]	0.75
HbA1c (mmol/mol)	220	36.0 [34.0-39.0]	37.0 [35.0-39.5]	36.0 [34.0-40.5]	36.0 [34.0-37.0]	37.0 [34.0-39.0]	0.42
Neuroimaging							
Microbleeds (yes,%)	231	18.1%	15.6%	24.2%	11.9%	21.2%	0.38
WM hypointensities (mm^3^)	231	1542.3 [859.3-3186.5]	1195.6 [774.0-2986.4]	1984.2 [1125.2-3694.1]	1791.2 [1073.3-2664.1]	932.1 [617.7-2116.2]	0.02^e, f^
HV/CTX	231	0.262 [0.240-0.282]	0.299 [0.289-0.312]	0.225 [0.217-0.233]	0.262 [0.252-0.274]	0.264 [0.259-0.270]	< 0.001^a, b, c, d, e^
BV/CSF	231	820.0 [699.5-971.1]	869.9 [720.9-1000.3]	798.7 [698.1-913.6]	709.3 [662.9-766.9]	1024.3 [953.2-1119.7]	< 0.001^b, c, d, e, f^
CSF biomarkers							
Aß42, pg/mL	231	633.0 [493.0-815.0]	689.0 [545.0-866.5]	604.5 [491.0-775.7]	575.0 [431.0-714.0]	718.0 [588.0-1025.0]	< 0.001^b, e, f^
Aß42/Aß40	231	0.053 [0.04-0.06]	0.058 [0.050-0.067]	0.049 [0.039-0.060]	0.051 [0.0442-0.058]	0.057 [0.048-0.066]	0.02^a, b, e^
p-tau, pg/ml	231	72.0 [52.0-100.0]	61.0 [53.5-78.5]	84.5 [57.0-120.0]	83.0 [54.0-110.0]	61.0 [48.0-79.5]	< 0.001^a, e, f^
t-tau, pg/ml	231	468.0 [325.5-643.0]	399.0 [308.5-572.5]	536.0 [357.2-770.7]	520.0 [357.0-660.0]	384.0 [310.0-486.0]	0.01^e, f^
NFL, pg/ml	231	1020.0 [755.0-1310.0]	910.0 [730.0-1300.0]	1125.0 [855.0-1360.0]	1120.0 [865.0-1395.0]	810.0 [640.0-975.0]	< 0.001^e, f^
t-tau/Aß42	231	0.75 [0.58-0.96]	0.69 [0.55-0.87]	0.78 [0.62-0.97]	0.83 [0.66-1.10]	0.66 [0.46-0.81]	< 0.001^b, e, f^
Disease stage							
SCI (%)		21.2%	23.5%	15.1%	11.9%	40.4%	<0.001^a, b, c, e, f^
MCI (%)	231	46.8%	43.1%	53.0%	37.3%	55.3%	
Dementia (%)		32.0%	33.3%	31.8%	50.7%	4.2%	

All quantitative data are represented with median and 1st interquartile and 3rd interquartile [Q1-Q3]. Letters indicate significant pairwise comparisons, after Dwass-Steel-Critchlow-Fligner (DSCF) or Bonferroni correction

WM hypointensities are presented as “raw” data and normalized for the ICV to assess differences across subtypes

^a^Cortical predominant versus limbic predominant

^b^Cortical predominant versus typical

^c^Cortical predominant versus minimal atrophy

^d^Limbic predominant versus typical

^e^Limbic predominant versus minimal atrophy

^f^Typical versus minimal atrophy

No significant differences were observed across subtypes for age (median: 61.0 years), education (median: 13.5 years), gender (overall 68.4% females), or APOE ε4 carriership (68.4%).

MoCA scores were highest in the minimal (median: 24.0) and limbic predominant (median: 23.0) groups, and lowest in the typical and cortical predominant subtypes (median: 18.0 and 21.0, respectively).

**Fig. 2:**
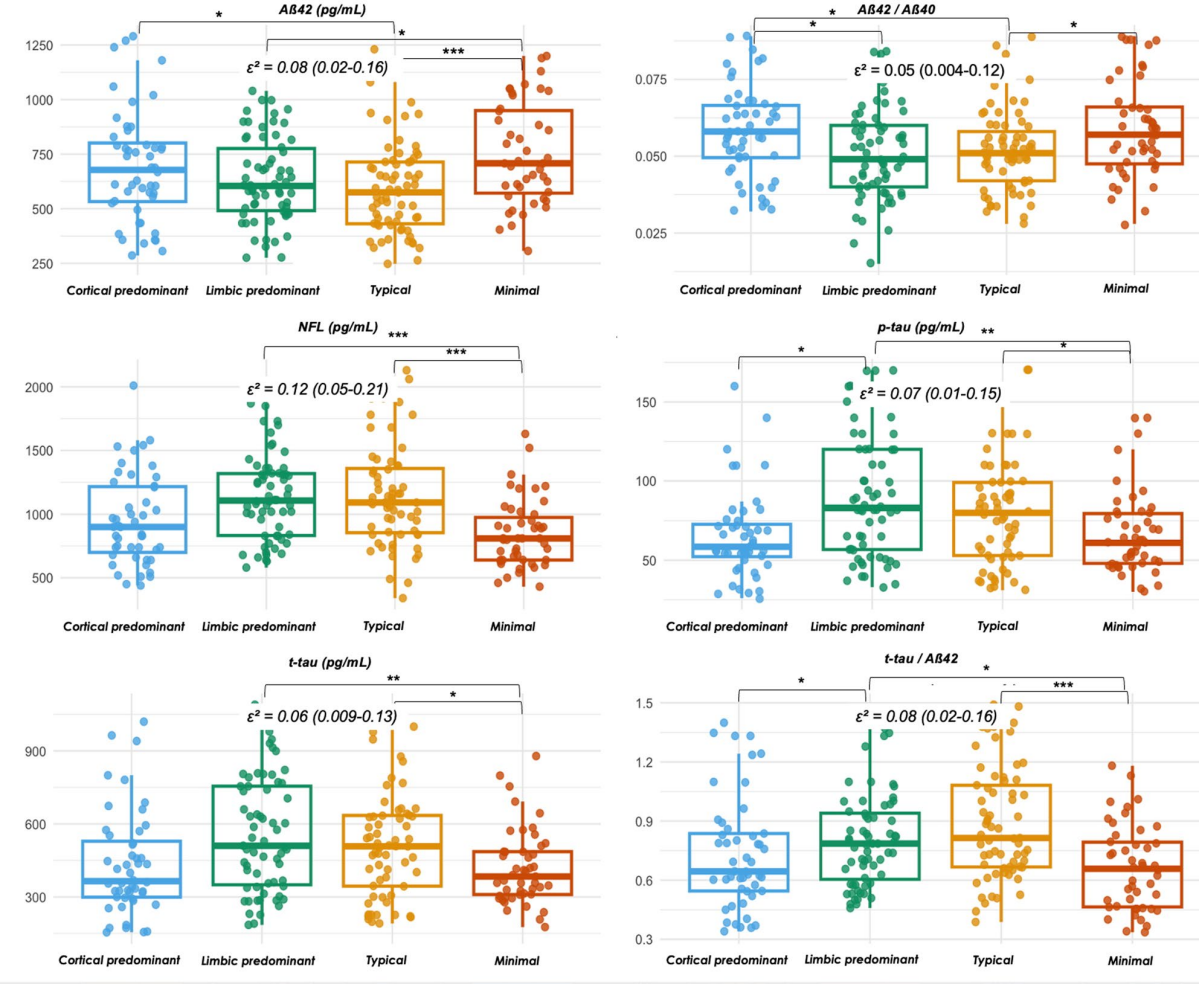
Comparison of CSF biomarkers among biological subtypes in the Aβ-positive cohort. Group differences in CSF biomarkers across MRI-based atrophy subtypes (cortical predominant, limbic predominant, typical, minimal atrophy). Boxplots show the median, interquartile range (IQR), and individual data points. Between-group comparisons were performed using the Kruskal–Wallis test with the Dwass–Steel–Critchlow–Fligner (DSCF) correction. Pairwise post hoc differences are indicated by brackets with significance levels (*p < 0.05, **p < 0.01, ***p < 0.001). Effect sizes are reported as epsilon squared (ε²) for each biomarker panel, with 95% confidence intervals

Neuropsychological testing revealed a trend of a greater episodic and visual memory impairment in the typical subtype. PHQ-9 scores were higher in the minimal atrophy group (median: 8.0; *p* = 0.02, η² = 0.07; 95% CI, 0.02–0.17). CSF biomarkers indicated higher AD-related pathology in the limbic predominant and typical subtypes, with lower Aβ42 and Aβ42/40 ratios (median: 0.049 and 0.051, respectively) higher p-tau levels (84.5 and 83.0 pg/mL) and t-tau/Aβ42 ratio (0.78 and 0.83) than the other subtypes (*p* < 0.05, ε² ≥ 0.05). Neurodegeneration markers, including NFL (1125.0 and 1120.0 pg/mL) and t-tau (536.0 and 520.0 pg/mL), were also elevated in these groups (*p* < 0.05, ε² ≥ 0.06) (Fig. 2). Additionally, the limbic predominant and typical subtypes exhibited greater WM hypointensity volumes (*p* = 0.02, ε² = 0.05; 95% CI, 0.004–0.13). No significant differences were observed in blood-based vascular risk markers across subtypes. Findings in individuals < 65 years were consistent also in the Aβ-positive cohort (Supplementary materials eTable 2).

### MRI-based atrophy subtype differences in the Aβ-negative cohort

Subtype differences in this cohort are summarized in the Supplementary Materials eTable 3. Cortical predominant and minimal atrophy subtypes were the most common (28.4% and 29.3%, respectively). Female representation was higher in the cortical predominant and minimal atrophy subtypes (67.3% and 62.6%, respectively; *p* = 0.006, Cramér’s V = 0.18; 95% CI 0.10–0.29). The typical subtype was the oldest (median age: 61.0 years), while the minimal atrophy group was the youngest (median age: 57.0 years; *p* = 0.01, ε² = 0.03; 95% CI, 0.001–0.07). MoCA scores (median: 25.0), most cognitive tests, and CSF biomarkers did not differ significantly across subtypes. Exceptions included higher NFL concentrations in the typical group (median: 820 pg/mL; *p* < 0.001, ε² = 0.04; 95% CI, 0.01–0.09) and greater WM hypointensity volumes in both the typical and limbic predominant subtypes (*p* < 0.001, ε² = 0.09; 95% CI, 0.05–0.17). APOE ε4 carriership did not differ significantly between groups.

### Continuous subtype dimensions in the Aβ-Positive cohort

**Fig. 3:**
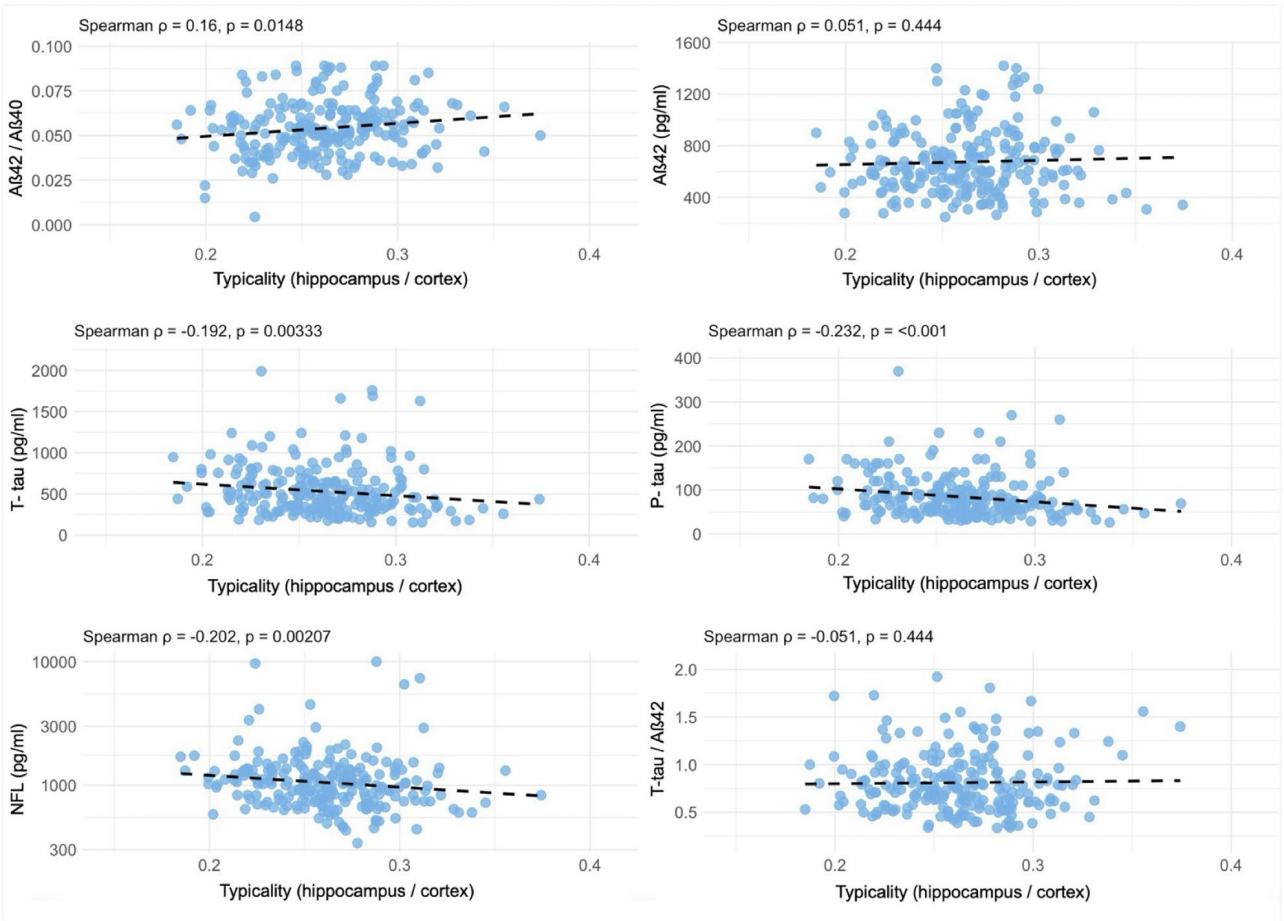
Scatterplots of correlation between typicality (hippocampus/cortex ratio) and CSF biomarkers in the Aβ-positive cohort. Correlation coefficients represent Spearman rho (ρ). p ≤ 0.05 was considered significant

**Fig. 4:**
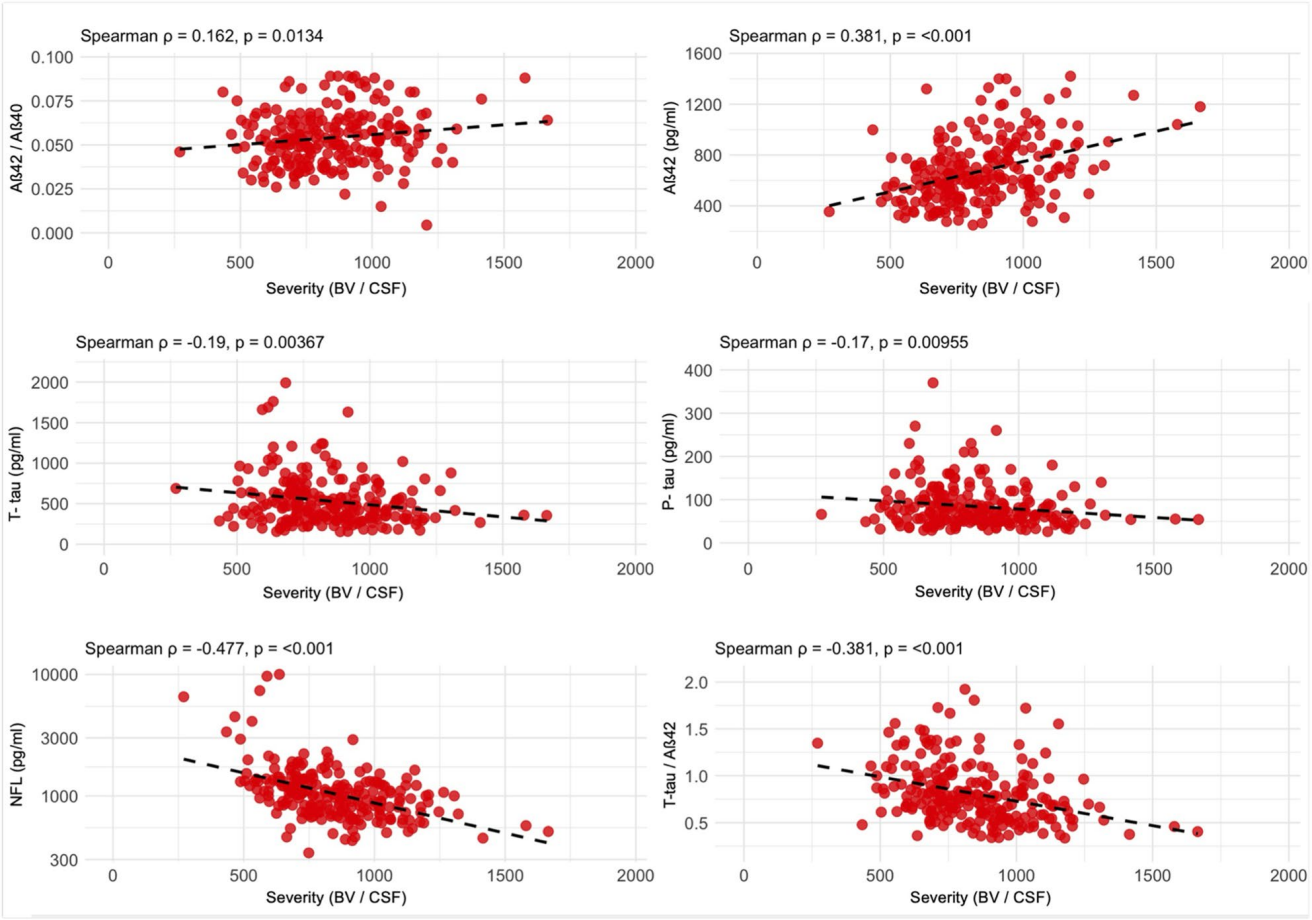
Scatterplots of correlation between severity (brain volume/CSF ratio) and CSF biomarkers in the Aβ-positive cohort. Correlation coefficients represent Spearman rho (ρ). p ≤ 0.05 was considered significant

Figures 3 and 4 show the Spearman correlation matrices for typicality (HV/CTX) and severity (BV/CSF). Typicality correlated positively with Aβ42/40 ratio (ρ = 0.16, p = 0.015), and negatively with t-tau (ρ = −0.192, p = 0.003), p-tau (ρ = −0.232, p < 0.001), NFL (ρ = −0.202, p = 0.002), and WM hypointensities (ρ = −0.142, p = 0.016). Severity showed positive associations with Aβ42/40 (ρ = 0.162, p = 0.013) and Aβ42 (ρ = 0.381, p < 0.001), and negative associations with t-tau (ρ = −0.19, p = 0.003), p-tau (ρ = −0.17, p = 0.01), NFL (ρ = −0.477, p < 0.001), the t-tau/Aβ42 ratio (ρ = −0.381, p < 0.001), and WM hypointensities (ρ = −0.432, p < 0.001).

Typicality correlated positively with Aβ42/40 ratio (ρ = 0.16, *p* = 0.015), and negatively with t-tau (ρ = −0.192, *p* = 0.003), p-tau (ρ = −0.232, *p* < 0.001), NFL (ρ = −0.202, *p* = 0.002), and WM hypointensities (ρ = −0.142, *p* = 0.016).

Severity showed positive associations with Aβ42/40 (ρ = 0.162, *p* = 0.013) and Aβ42 (ρ = 0.381, *p* < 0.001), and negative associations with t-tau (ρ = −0.19, *p* = 0.003), p-tau (ρ = −0.17, *p* = 0.01), NFL (ρ = −0.477, *p* < 0.001), the t-tau/Aβ42 ratio (ρ = −0.381, *p* < 0.001), and WM hypointensities (ρ = −0.432, *p* < 0.001).

### Anti-Aβ Treatment-eligible cohort characteristics

Among patients with available CSF data, 89 individuals (14.9%) met the eligibility criteria for anti-Aβ therapy. Subtypes were distributed as follows: limbic predominant (31.5%), typical (28.1%), cortical predominant (21.3%), and minimal atrophy (19.1%). No significant differences were observed across subtypes in APOE ε4 status or blood vascular-risk markers. However, NFL concentrations were higher in the limbic predominant group (median: 1105.0 pg/mL; *p* = 0.013). Typicality showed inverse associations with p-tau (ρ = − 0.253, *p* = 0.008), t-tau (ρ = − 0.223, *p* = 0.018), NFL (ρ = − 0.276, *p* = 0.004), and WM hypointensity volume (ρ = − 0.168, *p* = 0.05). Severity was also negatively correlated with NFL (ρ = − 0.444, *p* < 0.001) and WM hypointensity volume (ρ = − 0.455, *p* < 0.001).

## Discussion

Our study identified distinct MRI-based atrophy subtypes within a memory clinic cohort primarily composed of relatively young patients at early stages of cognitive impairment. These subtypes exhibited significant differences in demographic characteristics, CSF biomarkers, and cerebrovascular burden, reflecting biological heterogeneity. Notably, these differences persisted across the AD continuum and remained detectable even in patients eligible for anti-amyloid therapies, reinforcing the potential of MRI-based classification to support diagnosis and precision medicine in real-world settings.

Three main strengths of our study enhance its generalizability and contribution to the literature on brain atrophy heterogeneity in memory clinics: (I) The naturalistic study design ensures real-world applicability of our findings. (II) The volumetric classification integrated both categorical and continuous methods, grounded in established neuropathological models [6, 7]. This approach reduces the subjectivity of visual rating of atrophy while remaining more practical than unsupervised clustering [26]. (III) The large number of patients with CSF data enabled robust biological characterization and facilitated detailed comparisons across subtypes.

Our analysis revealed a higher prevalence of Aβ positivity in the limbic predominant and typical subtypes compared to the cortical predominant and minimal atrophy groups. This finding aligns with the known vulnerability of temporomesial regions in AD [46], but must be interpreted in the context of our relatively young cohort. Notably, subtypes sparing the hippocampus remain less frequent in AD, even in a relatively young population, primarily composed of patients with SCI or MCI.

The prevalence of APOE ε4 carriers, the strongest genetic risk factor for sporadic AD [47], was higher in subtypes characterized by hippocampal or diffuse atrophy, confirming previous findings in the AD literature [11, 48]. However, when restricting the analysis to Aβ-positive individuals, this trend was no longer statistically significant, although the prevalence remained higher in patients with the limbic predominant subtype. This is consistent with evidence suggesting that the impact of APOE ε4 may be attenuated in younger populations [49]. Moreover, most prior data derive from AD dementia cohort, while our study includes earlier stages such as SCI and MCI.

Female patients were more common in the minimal atrophy and cortical predominant subtypes, whereas males were more prevalent in the typical and limbic predominant groups. Prior studies have reported similar findings in patients with preserved metabolism on FDG-PET, corresponding to minimal atrophy [18]. However, our sex distribution for the cortical predominant and limbic predominant groups diverges from prior work [11], potentially due to our younger cohort and limited representation of advanced-stage cases. In older populations, female predominance in the limbic subtype has been linked to longer life expectancy, a pattern that may be less relevant at earlier stages. Previous studies in AD have suggested that, in patients with dementia, lower cognitive reserve may contribute to the emergence of clinical symptoms in the minimal atrophy subtype, despite the absence of marked structural changes [9].

In our cohort, educational attainment, a commonly used proxy of cognitive reserve, did not significantly differ across MRI-based atrophy subtypes. This finding does not provide direct support for the hypothesis that lower cognitive reserve, as indexed by education alone, drives symptom onset in the minimal atrophy subtype. Importantly, our sample included younger individuals, often at earlier clinical stages compared with those examined in previous studies, and education may incompletely capture the multidimensional nature of cognitive reserve in this context. Alternative mechanisms may contribute to cognitive complaints in this group. Patients classified within the minimal atrophy subtype showed higher levels of depressive symptoms on the PHQ-9, and most were in the SCI or MCI stages. This suggests that depressive symptoms could partially account for both subjective and mild objective cognitive impairment, potentially influencing the clinical presentation of this subtype.

Cognitive profiles varied across subtypes. The typical and limbic predominant groups showed greater episodic memory impairment, whereas the cortical predominant individuals exhibited poorer visuospatial performance among Aβ-positive individuals. The minimal atrophy subtype generally performed better, consistent with earlier disease stages. The typical subtype also included a higher proportion of demented patients.

In terms of cerebrovascular pathology, subtypes with diffuse or hippocampal atrophy had higher WM hypointensities and elevated serum homocysteine levels, aligning with evidence linking homocysteine to both global cortical and hippocampal atrophy [50], and WM damage [51]. The greater cerebrovascular burden in the typical and limbic predominant groups supports prior findings in AD cohorts [11].

To examine the biological correlates of MRI-based atrophy subtypes, we stratified the cohort by Aβ status and focused specifically on Aβ-positive patients. Whether AD subtypes differ meaningfully in CSF biomarkers in relatively young patients in a real-world setting remains insufficiently examined. In our study, significant differences emerged within the Aβ-positive group. Using both a categorical approach (four subtypes) and a continuous approach (assessing typicality and severity), the limbic predominant and typical AD subtypes showed higher levels of AD-related biomarkers, including lower Aβ42 (or Aβ42/Aβ40 ratios), elevated p-tau and t-tau/Aβ42 ratio, compared to the cortical predominant and minimal atrophy subtypes. These findings suggest a higher overall amyloid and tau pathology burden in the limbic predominant and typical subtypes.

While tau differences across subtypes are well established, fewer studies have found consistent differences in amyloid levels [19, 48, 52].

Our relatively young cohort may have captured purer forms of typical and limbic AD, less influenced by co-pathologies such as MAPT H1H1, hippocampal sclerosis, or TDP-43, which are more common in older populations [11]. The similarity in biomarker profiles supports the view that the typical and limbic patterns may represent a continuum, with limbic predominant potentially evolving toward typical AD [17].

In addition to AD-related specific biomarkers, markers of non-specific neurodegeneration, including NFL, t-tau, and WM hypointensities, were elevated in the limbic predominant and typical subtypes. Although WM hypointensities, used as a proxy for microvascular damage [53], remained elevated in Aβ-positive patients, homocysteine levels did not differ significantly across subtypes.

While our findings highlight greater AD-related biomarker abnormalities, non-specific neurodegeneration, and cerebrovascular burden in the limbic predominant and typical subtypes, the biological underpinnings of the cortical predominant and minimal atrophy subtypes remain less well defined. The cortical predominant subtype has been associated with faster progression and distinct NFT patterns [54]. Our findings support its distinctiveness, although current biomarkers may not fully capture its pathophysiology, which could involve alternative pathways, such as neuroinflammatory changes or coexisting Lewy body pathology [11, 54].

Two potential mechanisms may explain the minimal atrophy subtype. First, studies suggest increased α-synuclein in this group, contributing to symptoms without overt atrophy [55]. This is consistent with neuropathological studies identifying α-synuclein accumulation in minimal atrophy AD cases [16]. Second, minimal atrophy AD may represent an earlier disease stage in which depressive symptoms accelerate the onset of cognitive decline. Over time, this subtype may progress into more overt atrophy patterns [17].

The naturalistic design of our study allowed us to analyze a considerable Aβ-negative group. To our knowledge, the existence of biologically defined subtypes within Aβ-negative cohorts in memory clinics has never been systematically investigated, and it remains unclear whether such subtypes truly reflect distinct pathophysiological processes. Although in-depth characterization of this group was beyond the scope of the current work, no significant differences in biomarkers were observed across the four subtypes, except for higher NFL levels and greater cerebrovascular burden in the typical subtype compared to the minimal atrophy group. Additionally, no significant variation was detected along the typicality axis. These findings support the hypothesis that observed atrophy-related differences, are primarily AD-related particularly in the typicality.

We also explored whether these biological distinctions persist in individuals eligible for anti-Aβ therapies based on current lecanemab guidelines [27]. Only 14.9% met the criteria, reflecting stringent recommendations. Although the small sample size limited the power to detect subtype differences using a categorical framework, a consistent pattern emerged along continuous measures of atrophy: increasing typicality toward hippocampal atrophy was linked to higher p-tau, t-tau, NFL, and cerebrovascular burden, and increasing severity was associated with rising NFL and cerebrovascular pathology.

Understanding how demographic, genetic, and biomarker profiles differ across AD subtypes is critical for advancing precision medicine. As disease-modifying therapies emerge [56], more refined biological subtyping of AD could optimize treatment outcomes through personalized strategies [57].

In summary, MRI-based subtyping captured meaningful biological differences even in a relatively young real-world population. These distinctions remained evident in both Aβ-positive and treatment-eligible individuals, highlighting the clinical utility of atrophy-based classification for diagnosis and personalized care in AD.

This study has limitations. First, the relatively small number of patients older than 65 years restricts generalizability to late-onset AD, reflecting both the Karolinska clinic’s focus on younger patients and Swedish referral patterns. Nevertheless, this relatively young-onset population remains undercharacterized, and our findings provide important insights into this group. Second, only a minority of patients fulfilled current treatment eligibility criteria, in line with recent epidemiological evidence [28, 58], thereby limiting statistical power for subgroup analyses. Third, the cross-sectional design captures only a static picture of atrophy subtypes, precluding conclusions regarding their temporal evolution. Longitudinal studies are warranted to elucidate trajectories of subtype-specific progression. Finally, the use of cohort-specific percentile-based cut-offs facilitated internal stratification but limits the external generalizability of our results, particularly to late-onset populations.

Nonetheless, our study represents one of the most comprehensive cross-sectional analyses of MRI-based subtypes conducted within a single cohort, combining a substantial sample size with extensive CSF biomarker characterization. Importantly, focusing on a single cohort also mitigates potential bias arising from heterogeneity in MRI acquisition and processing protocols, which is a common limitation of multi-cohort studies.

## Conclusion

This study offers novel insights into MRI-based subtypes in a real-world, relatively young memory clinic population. Subtypes differed in demographics, biomarkers, and cerebrovascular burden, reinforcing the biological heterogeneity of naturalistic memory clinics. Biologically informed subtyping offers a promising avenue to enhance diagnostic accuracy and personalized therapeutic strategies in AD.

## Supplementary Information

Supplementary material 1: eTable.1.docx. Summary of the demographic, cognitive assessment, neuroimaging, and CSF biomarkers of the global cohort under 65. eTable.2.docx. Summary of the demographic, cognitive assessment, neuroimaging, and CSF biomarkers of the Aβ-positive cohort under 65. eTable.3.docx. Summary of the demographic, cognitive assessment, neuroimaging, and CSF biomarkers of the Aβ-negative cohort.

## Data Availability

We are open to requests for data collected in this study. Proposals must include a detailed study plan outlining the research question, analysis plan, and specific data needed. Only deidentified data and the data dictionary are shared. Analyses will be conducted in collaboration with our team, and data access will be subject to the GEDOC legal framework. A formal data access agreement will be needed.

## Abbreviations

AD: Alzheimer’s disease
APOE: Apolipoprotein E
Aβ: Amyloid-β
Aβ42/40: Amyloid-β42/40 ratio
ANCOVA: Analysis of covariance
ANOVA: Analysis of variance
BV/CSF: Brain volume to cerebrospinal fluid index
CSF: Cerebrospinal fluid
DSCF: Dwass–Steel–Critchlow–Fligner
EOAD: Early-onset Alzheimer’s disease
GLM: General linear model
HV/CTX: Hippocampal-to-cortical volume ratio
ICD-10: International Classification of Diseases, 10th revision
ICV: Intracranial volume
IQR: Interquartile range
MCI: Mild cognitive impairment
MMSE: Mini-Mental State Examination
MoCA: Montreal Cognitive Assessment
MRI: Magnetic resonance imaging
NFT: Neurofibrillary tangles
NFL: Neurofilament light chain
PCA: Posterior cortical atrophy
PPA: Primary progressive aphasia
PHQ-9: Patient Health Questionnaire-9
p-tau181: Phosphorylated tau at threonine 181
RAVLT: Rey Auditory Verbal Learning Test
RCF: Rey Complex Figure Test
SCI/SCD: Subjective cognitive impairment/Subjective cognitive decline
SWI: Susceptibility-weighted imaging
T-tau: Total tau
WAIS-IV: Wechsler Adult Intelligence Scale, 4th edition
WM: White matter
