# Supplementary material for: MRI-based atrophy subtypes in a young memory clinic cohort: associations with clinical and biomarker profiles
Source: Alzheimers Res Ther. 2026 Feb 10;18:35. doi: 10.1186/s13195-026-01972-2 (PMC12895802; doi:10.1186/s13195-026-01972-2)
Supplement: Supplementary file 1 — Supplementary material 1: eTable.1.docx. Summary of the demographic, cognitive assessment, neuroimaging, and CSF biomarkers of the global cohort under 65. eTable.2.docx. Summary of the demographic, cognitive assessment, neuroimaging, and CSF biomarkers of the Aβ-positive cohort under 65. eTable.3.docx. Summary of the demographic, cognitive assessment, neuroimaging, and CSF biomarkers of the Aβ-negative cohort. [file 13195_2026_1972_MOESM1_ESM.docx]

|  | N | All | Cortical predominant | Limbic predominant | Typical | Minimal atrophy | Global P-value FDR-corrected and pairwise comparisons |
| --- | --- | --- | --- | --- | --- | --- | --- |
| Sample size | 691 | 691 | 181 | 161 | 165 | 184 | - |
| Demographic variables  Age (years)  Gender (female, %)  Education (years)  APOE (ε4 +/− or +/+, %) | 691  691  548  608 | 59.0 [55.0-62.0]  57.9%  13.0 [11.0-15.5]  44.6% | 59.9 [55.0-62.0]  69.6%  13.0 [11.7-15.5]  36.8% | 60.0 [55.0-62.0]  41.6%  13.0 [11.0-16.0]  55.9% | 60.0 [56.0-62.0]  50.9%  13.0 [11.0-15.0]  45.6% | 57.0 [54.0-61.0]  67.4%  12.5 [11.0-15.5]  42.1% | 0.01 ^f^  < 0.001^a, b, e, f^  0.78  0.03 ^a^ |
| Cognitive assessment  MoCA  MMSE  RAVLT immediate recall  RAVLT delayed recall  RCF copy  RCF delayed recall  WAIS-IV coding  PHQ-9 | 552  421  425  423  408  408  408  516 | 24.0 [21.0-27.0]  27.0 [24.0-29.0]  44.0 [33.0-51.0]  10.0 [6.0-13.0]  33.0 [30.5-34.0]  15.0 [9.0-21.5]  50.5 [39.0-62.0]  7.0 [3.0-12.0] | 24.0 [20.0-26.0]  26.0 [23.0-28.5]  44.0 [32.0-51.0]  10.0 [7.0-12.0]  33.0 [30.6-35.0]  15.0 [9.0-19.5]  48.0 [37.0-59.5]  7.3 [2.9-12.0] | 24.0 [21.7-26.0]  27.3 [24.0-29.0]  39.5 [31.7-49.2]  8.0 [4.0-12.0]  33.0 [29.7-34.0]  14.2 [7.0-21.1]  53.0 [43.0-63.0]  6.0 [2.0-12.0] | 24.0 [19.0-27.0]  27.0 [23.0-29.0]  38.0 [30.0-49.2]  8.0 [3.7-12.0]  32.0 [32.0-34.0]  14.0 [6.5-20.0]  45.0 [34.7-61.0]  6.5 [2.0-10.0d] | 25.0 [22.0-27.0]  27.0 [25.0-29.0]  47.0 [37.2-55.0]  11.0 [9.0-13.0]  33.0 [31.0-34.0]  18.0 [12.7-23.0]  54.0 [44.5-65.0]  8.0 [4.0-13.0] | 0.06  0.10  0.01 ^e, f^  < 0.001^a, b, e, f^  0.29  < 0.001 ^e, f^  0.13  0.15 |
| Blood biomarkers  Homocysteine (µmol/L)  LDL-cholesterol (mmol/L)  HbA1c (mmol/mol) | 644  599  627 | 12.0 [10.0-15.0]  3.0 [2.5-3.7]  36.0 [34.0-39.0] | 12.0 [9.9-14.0]  2.9 [2.3-3.7]  37.0 [34.0-39.0] | 13.0 [10.0-15.0]  3.0 [2.5-3.6]  36.0 [34.0-39.0] | 13.0 [11.0-16.0]  3.0 [2.4-3.7]  36.0 [34.0-39.0] | 12.0 [9.9-14.0]  3.2 [2.6-3.8]  36.0 [34.0-38.0] | 0.01^b, f^  0.26  0.42 |
| Neuroimaging biomarkers  HV/CTX  BV/CSF  WM hypointensities (mm^3^) | 691  691  691 | 0.26 [0.24-0.28]  908.9 [752.9-1051.5]  1164 [725.0-2254.6] | 0.30 [0.29-0.31]  944.4 [820.0-1104.9]  862.7 [594.8-1531.0] | 0.23 [0.21-0.24]  869.3 [714.0-987.4]  1561.7 [969.3-3178.8] | 0.26 [0.25-0.27]  738.9 [670.5-803.6]  1599.9 [1059.5-3273.7] | 0.26 [0.25-0.27]  1030.7 [968.3-1175.3]  916.9 [625.1-1642.5] | < 0.001^a, b, c, d, e^  < 0.001^a, b, c, d, e, f^  < 0.001^a, b, e, f^ |
| CSF biomarkers  Aß42, pg/mL  Aß42/Aß40  p-tau, pg/ml  t-tau, pg/ml  NFL, pg/ml  t-tau/Aß42  Amyloid status (A+, N and %) | 517  517  517  517  515  517  517 | 927.0 [665.0-1200.0]  0.09 [0.05-0.10]  40.0 [30.0-59.0]  285.0 [202.0-416.0]  750.0 [590.0-1050.0]  0.51 [0.39-0.71]  185 (35.8%) | 990.0 [772.0-1290.0]  0.09 [0.07-0.10]  39.0 [27.0-54.0]  268.0 [188.0-357.0]  720.0 [550.0-940.0]  0.48 [0.36-0.61]  43 (30.5%) | 825.0 [608.2-996.5]  0.08 [0.05-0.10]  42.5 [31.0-81.7]  296.0 [223.0-514.5]  835.0 [627.5-1247.5]  0.57 [0.47-0.78]  52 (44.0%) | 842.0 [548.0-1130.0]  0.07 [0.05-0.10]  42.0 [31.0-72.0]  302.0 [216.0-512.0]  900.0 [670.0-1160.0]  0.56 [0.42-0.87]  51 (43.6%%) | 1050.0 [768.5-1277.5]  0.10 [0.07-0.10]  40.0 [30.0-50.7]  273.5 [197.5-358.0]  670.0 [542.5-877.5]  0.45 [0.37-0.62]  39 (28.2%) | < 0.001^a, b, e, f^  < 0.001 ^a, b, e, f^  0.04^a, e^  0.01^a, b, e, f^  < 0.001^a, b, e, f^  < 0.001^a, b, e, f^  0.03^a, b, e, f^ |
| Disease stage  SCD (%)  MCI (%)  Dementia (%) | 691  691  691 | 41.1%  44.7%  14.2% | 44.1%  43.7%  12.2% | 31.6%  52.9%  15.5% | 36.8%  38.3%  24.9% | 50.0%  44.5%  5.5% | < 0.001^e, f^ |

**eTab.1: Summary of the demographic, cognitive assessment, neuroimaging, and CSF biomarkers of the global cohort under 65.**

All quantitative data are represented with median and 1st interquartile and 3rd interquartile [Q1-Q3]. Letters indicate significant pairwise comparisons, after Dwass-Steel-Critchlow-Fligner (DSCF) or Bonferroni correction: a, cortical predominant versus limbic predominant; b, cortical predominant versus typical; c, cortical predominant versus minimal atrophy; d, limbic predominant versus typical; e, limbic predominant versus minimal atrophy; f, typical versus minimal atrophy.

WM hypointensities are presented as “raw” data and normalized for the ICV to assess differences across subtypes.

Abbreviations: APOE, apolipoprotein E; MoCA, Montreal Cognitive Assessment; MMSE, Mini-Mental State Examination; RALVT, Rey Auditory Verbal Learning Test; RCF, Rey Complex Figure Test; WAIS, Wechsler Adult Intelligence Scale; PHQ-9, Patient Health Questionnaire; HV/CTX, hippocampal volume/cortex ratio; BV/CSF, brain volume/cerebrospinal fluid ratio; WM: white matter; Aß, amyloid-beta; NFL, neurofilament light chain; SCD, subjective cognitive decline; MCI, mild cognitive impairment.

|  | N | All | Cortical predominant | Limbic predominant | Typical | Minimal atrophy | Global P-value FDR-corrected and pairwise comparisons |
| --- | --- | --- | --- | --- | --- | --- | --- |
| Sample size | 185 | 185 | 43 | 52 | 51 | 39 | - |
| Demographic variables  Age (years)  Gender (female, %)  Education (years)  APOE (ε4 +/− or +/+, %) | 185  185  146  180 | 60.0 [57.0-62.0]  61.1%  13.0 [11.0-15.0]  67.8% | 61.0 [58.0-63.0]  72.1%  13.5 [12.0-16.70]  67.4% | 60.0 [58.0-62.0]  48.0%  12.5 [10.0-15.0]  74.5% | 59.0 [56.0-62.0]  58.2%  13.0 [11.0-15.0]  61.7% | 58.0 [55.5-61.0]  69.2%  13.0 [10.5-15.0]  66.6% | 0.16  0.1  0.63  0.61 |
| Cognitive assessment  MoCA  MMSE  RAVLT immediate recall  RAVLT delayed recall  RCF copy  RCF delayed recall  WAIS-IV coding  PHQ-9 | 153  124  117  116  111  111  112  136 | 23.0 [17.0-25.0]  26.0 [22.5-28.2]  35.0 [28.0-47.0]  7.0 [3.0-11.0]  33.0 [30.0-35.0]  12.5 [6.0-18.0]  47.0 [35.0-59.2]  5.0 [2.0-10.2] | 23.0 [18.2-26.0]  24.0 [22.3-28.0]  36.0 [30.0-50.0]  7.5 [5.5-12.0]  32.0 [27.0-35.0]  12.0 [3.5-18.5]  44.0 [31.0-49.0]  7.0 [2.2-11.7] | 23.0 [19.0-24.5]  28.0 [24.0-29.0]  38.0 [29.5-47.2]  5.5 [3.0-10.5]  33.7 [30.7-34.2]  11.0 [7.5-19.0]  55.0 [41.5-62.0]  4.0 [1.0-9.2] | 18.5 [15.0-24.0]  23.0 [20.0-27.0]  29.0 [20.7-37.2]  4.0 [2.0-8.0]  32.5 [29.0-34.7]  7.0 [3.5-15.2]  36.0 [32.0-51.0]  3.0 [1.6-8.0] | 24.0 [22.0-25.0]  27.0 [24.7-29.0]  41.0 [32.5-49.5]  9.0 [4.7-12.2]  33.0 [31.0-35.0]  14.5 [11.5-18.0]  51.0 [44.0-64.0]  8.0 [4.0-13.0] | 0.03^f^  0.02^f^  0.03^f^  0.02^f^  0.29  0.1  0.12  0.02^f^ |
| Blood biomarkers  Homocysteine (µmol/L)  LDL-cholesterol (mmol/L)  HbA1c (mmol/mol) | 181  170  177 | 13.0 [10.0-15.0]  3.3 [2.6-3.9]  36.0 [34.0-38.0] | 13.0 [10.0-15.0]  3.1 [2.4-3.6]  36.5 [35.0-39.0] | 13.0 [12.0-16.0]  3.3 [2.7-3.8]  35.5 [34.0-37.2] | 13.0 [11.0-15.0]  3.1 [2.6-3.8]  36.0 [34.0-37.0] | 11.0 [9.2-15.0]  3.6 [2.6-4.1]  37.0 [33.2-38.7] | 0.30  0.56  0.35 |
| Neuroimaging  Microbleeds (yes,%)  WM hypointensities (mm^3^)  HV/CTX  BV/CSF | 185  185  185  185 | 16.2%  1336.9 [790.1-2621.0]  0.26 [0.24-0.28]  860.0 [710.4-1009.5] | 11.6%  1088.4 [750.1-2554.8]  0.30 [0.28-0.31]  888.1 [771.4-1010.5] | 21.1%  1745.7 [1041.1-3283.1]  0.22 [0.21-0.23]  819.2 [711.5-949.8] | 11.7%  1535.4 [1059.6-2589.8]  0.26 [0.25-0.27]  704.5 [656.3-766.9] | 20.5%  860.6 [588.4-1770.7]  0.26 [0.25-0.27]  1026.8 [959.4-1136.7] | 0.30  0.01^e, f^  < 0.001^a, b, c, d, e^  < 0.001^b, c, d, e, f^ |
| CSF biomarkers  Aß42, pg/mL  Aß42/Aß40  p-tau, pg/ml  t-tau, pg/ml  NFL, pg/ml  t-tau/Aß42 | 185  185  185  185  185  185 | 636.0 [495.0-829.0]  0.054 [0.04-0.06]  71.0 [52.0-100.0]  458.0 [317.0-631.0]  980 [730.0-1230.0]  0.75 [0.57-0.96] | 741.0 [572.5-876.0]  0.058 [0.050-0.068]  59.0 [53.5-75.5]  369.0 [308.5-518.0]  900.0 [700.0-1215.0]  0.64 [0.54-0.83] | 617.0 [502.7-789.7]  0.051 [0.041-0.060]  84.5 [59.2-120.0]  536.0 [364.2-758.0]  1100 [830.0-1310.0]  0.77 [0.60-0.94] | 539.0 [431.0-685.5]  0.050 [0.042-0.056]  79.0 [53.0-110.0]  512.0 [325.0-660.0]  1080.0 [840.0-1345.0]  0.88 [0.69-1.10] | 708.0 [588.0-1035.0]  0.059 [0.048-0.067]  59.0 [47.0-77.5]  368.0 [306.5-498.0]  770.0 [635.0-930.0]  0.67 [0.46-0.81] | < 0.001^b, e, f^  0.01 ^b^  0.007^a, e, f^  0.01^a, e^  < 0.001^e, f^  < 0.001^b, e, f^ |
| Disease stage  SCD (%)  MCI (%)  Dementia (%) | 185 | 22.7%  48.1%  29.2% | 27.9%  44.1%  27.9% | 17.3%  55.7%  26.9% | 11.7%  37.2%  50.9% | 38.4%  56.4%  5.1% | <0.001^b, e, f^ |

**eTab.2: Summary of the demographic, cognitive assessment, neuroimaging, and CSF biomarkers of the Aβ-positive cohort under 65.**

All quantitative data are represented with median and 1st interquartile and 3rd interquartile [Q1-Q3]. Letters indicate significant pairwise comparisons, after Dwass-Steel-Critchlow-Fligner (DSCF) or Bonferroni correction: a, cortical predominant versus limbic predominant; b, cortical predominant versus typical; c, cortical predominant versus minimal atrophy; d, limbic predominant versus typical; e, limbic predominant versus minimal atrophy; f, typical versus minimal atrophy.

WM hypointensities are presented as “raw” data and normalized for the ICV to assess differences across subtypes.

Abbreviations: APOE, apolipoprotein E; MoCA, Montreal Cognitive Assessment; MMSE, Mini-Mental State Examination; RALVT, Rey Auditory Verbal Learning Test; RCF, Rey Complex Figure Test; WAIS, Wechsler Adult Intelligence Scale; PHQ-9, Patient Health Questionnaire; HV/CTX, hippocampal volume/cortex ratio; BV/CSF, brain volume/cerebrospinal fluid ratio; WM: white matter; Aß, amyloid-beta; NFL, neurofilament light chain; SCD, subjective cognitive decline; MCI, mild cognitive impairment.

|  | N | All | Cortical predominant | Limbic predominant | Typical | Minimal atrophy | Global P-value FDR-corrected and pairwise comparisons |
| --- | --- | --- | --- | --- | --- | --- | --- |
| Sample size | 365 | 365 | 104 | 77 | 77 | 107 | - |
| Demographic variables  Age (years)  Gender (female, %)  Education (years)  APOE (ε4 +/− or +/+, %) | 365  365  297  355 | 59.0 [55.0-62.0]  57.5%  13.0 [11.0-16.0]  32.9% | 58.0 [54.0-62.0]  67.3%  13.7 [12.0-15.0]  29.7% | 60.0 [56.0-62.0]  44.1%  13.0 [11.0-16.0]  42.1% | 61.0 [57.0-63.0]  50.6%  13.0 [11.6-15.3]  32% | 57.0 [54.5-61.0]  62.6%  13.0 [11.0-16.0]  30% | 0.01^f^  0.006^a^  0.99  0.28 |
| Cognitive assessment  MoCA  MMSE  RAVLT immediate recall  RAVLT delayed recall  RCF copy  RCF delayed recall  WAIS-IV coding  PHQ-9 | 296  229  228  227  224  224  224  283 | 25.0 [22.0-27.0]  28.0 [25.0-29.0]  46.0 [37.0-51.2]  11.0 [7.0-13.0]  33.0 [31.0-34.0]  17.0 [11.8-22.0]  55.0 [41.7-63.2]  7.0 [3.0-11.0] | 24.0 [21.0-26.0]  27.0 [24.0-29.0]  44.5 [36.0-49.0]  10.0 [7.0-12.0]  33.0 [31.0-35.0]  15.0 [11.0-19.5]  51.0 [40.0-61.0]  7.0 [3.0-11.0] | 25.0 [22.0-27.0]  29.0 [27.0-29.0]  41.0 [34.0-51.0]  9.0 [5.2-12.7]  33.0 [29.7-35.0]  15.2 [6.5-20.3]  55.0 [44.0-64.0]  6.0 [1.0-12.0] | 25.0 [21.0-28.0]  29.0 [27.0-29.0]  47.0 [35.0-52.5]  10.5 [6.0-13.2]  32.5 [31.0-34.0]  19.0 [11.5-22.5]  52.0 [37.5-62.5]  6.7 [3.0-9.0] | 25.0 [23.0-27.1]  28.0 [25.0-29.0]  48.5 [44.0-55.0]  11.0 [9.5-13.0]  33.0 [31.0-34.0]  19.0 [14.0-24.0]  56.0 [43.0-65.0]  7.0 [3.0-12.5] | 0.19  0.26  0.05  0.01^e^  0.85  0.04 ^e^  0.71  0.52 |
| Blood biomarkers  Homocysteine (µmol/L)  LDL-cholesterol (mmol/L)  HbA1c (mmol/mol) | 348  327  177 | 12.0 [10.0-14.0]  3.0 [2.4-3.7]  36.0 [34.0-39.0] | 11.0 [9.7-14.0]  2.9 [2.0-3.7]  36.5 [34.0-39.0] | 12.0 [10.0-14.0]  2.8 [2.3-3.5]  36.0 [33.0-39.0] | 13.0 [11.0-15.0]  2.9 [2.4-3.8]  37.0 [34.0-39.0] | 12.0 [10.0-14.0]  3.1 [2.6-3.7]  36.0 [34.0-38.0] | 0.13  0.48  0.42 |
| Neuroimaging  Microbleeds (yes,%)  WM hypointensities (mm^3^)  HV/CTX  BV/CSF | 360  365  365  365 | 13.3%  1033.7 [677.7-2164.9]  0.26 [0.24-0.28]  932.8 [787.6-1067.6] | 13.8%  785.7 [511.5-1322.9]  0.29 [0.29-0.30]  988.4 [835.4-1098.9] | 12.9%  1355.5 [511.5-1322.9]  0.23 [0.21-0.24]  906.1 [744.9-1049.4] | 17.3%  1662.3 [1034.1-2824.5]  0.26 [0.25-0.27]  760.2 [698.5-832.1] | 10.2%  915.5 [646.1-1576.4]  0.26 [0.25-0.27]  1021.3 [944.4-1150.5] | 0.58  <0.001^a, b, e, f^  < 0.001 ^a, b, c, d, e^  < 0.001^b, c, d, e, f^ |
| CSF biomarkers  Aß42, pg/mL  Aß42/Aß40  p-tau, pg/ml  t-tau, pg/ml  NFL, pg/ml  t-tau/Aß42 | 365  365  365  365  363  365 | 1080.0 [875.0-1320.0]  0.10 [0.09-0.11]  33.0 [26.0-42.0]  237.0 [185.0-308.0]  700 [550.0-965.0]  0.44 [0.36-0.54] | 1115.0 [893.2-1425.0]  0.10 [0.09-0.11]  33.0 [25.0-40.0]  227.0 [178.0-292.2]  685.0 [517.5-822.5]  0.42 [0.33-0.53] | 988.0 [827.0-1320.0]  0.10 [0.09-0.010]  34.0 [28.0-42.0]  249.0 [195.0-308.0]  720.0 [545.0-1115.0]  0.48 [0.36-0.57] | 1040.0 [842.0-1250.0]  0.10 [0.09-0.011]  32.0 [25.0-42.0]  251.0 [191.0-324.0]  820.0 [640.0-1160.0]  0.45 [0.38-0.56] | 1160.0 [910.0-1345.0]  0.10 [0.09-0.011]  32.0 [27.0-44.0]  221.0 [186.0-312.5]  650.0 [530.0-835.0]  0.41 [0.35-0.52] | 0.07  0.60  0.70  0.36  < 0.001^b, e^  0.07 |
| Disease stage  SCD (%)  MCI (%)  Dementia (%) | 365 | 52.9%  38.9%  8.2% | 51.9%  40.4%  7.7% | 45.4%  45.5%  9.1% | 58.5%  31.2%  10.3% | 55.1%  38.4%  6.5% | 0.62 |

**eTab.3: Summary of the demographic, cognitive assessment, neuroimaging, and CSF biomarkers of the Aβ-negative cohort.**

All quantitative data are represented with median and 1st interquartile and 3rd interquartile [Q1-Q3]. Letters indicate significant pairwise comparisons, after Dwass-Steel-Critchlow-Fligner (DSCF) or Bonferroni correction: a, cortical predominant versus limbic predominant; b, cortical predominant versus typical; c, cortical predominant versus minimal atrophy; d, limbic predominant versus typical; e, limbic predominant versus minimal atrophy; f, typical versus minimal atrophy.

WM hypointensities are presented as “raw” data and normalized for the ICV to assess differences across subtypes.

Abbreviations: APOE, apolipoprotein E; MoCA, Montreal Cognitive Assessment; MMSE, Mini-Mental State Examination; RALVT, Rey Auditory Verbal Learning Test; RCF, Rey Complex Figure Test; WAIS, Wechsler Adult Intelligence Scale; PHQ-9, Patient Health Questionnaire; HV/CTX, hippocampal volume/cortex ratio; BV/CSF, brain volume/cerebrospinal fluid ratio; WM: white matter; Aß, amyloid-beta; NFL, neurofilament light chain; SCD, subjective cognitive decline; MCI, mild cognitive impairment.
